# The Temperature Dependence of Sleep

**DOI:** 10.3389/fnins.2019.00336

**Published:** 2019-04-24

**Authors:** Edward C. Harding, Nicholas P. Franks, William Wisden

**Affiliations:** ^1^Department of Life Sciences, Imperial College London, London, United Kingdom; ^2^Centre for Neurotechnology, Imperial College London, London, United Kingdom; ^3^UK Dementia Research Institute, Imperial College London, London, United Kingdom

**Keywords:** sleep-wake cycle, thermoregulation, thermoregulatory behaviour, circadian, preoptic area, anterior hypothalamus, energy balance, nesting

## Abstract

Mammals have evolved a range of behavioural and neurological mechanisms that coordinate cycles of thermoregulation and sleep. Whether diurnal or nocturnal, sleep onset and a reduction in core temperature occur together. Non-rapid eye movement (NREM) sleep episodes are also accompanied by core and brain cooling. Thermoregulatory behaviours, like nest building and curling up, accompany this circadian temperature decline in preparation for sleeping. This could be a matter of simply comfort as animals seek warmth to compensate for lower temperatures. However, in both humans and other mammals, direct skin warming can shorten sleep-latency and promote NREM sleep. We discuss the evidence that body cooling and sleep are more fundamentally connected and that thermoregulatory behaviours, prior to sleep, form warm microclimates that accelerate NREM directly through neuronal circuits. Paradoxically, this warmth might also induce vasodilation and body cooling. In this way, warmth seeking and nesting behaviour might enhance the circadian cycle by activating specific circuits that link NREM initiation to body cooling. We suggest that these circuits explain why NREM onset is most likely when core temperature is at its steepest rate of decline and why transitions to NREM are accompanied by a decrease in brain temperature. This connection may have implications for energy homeostasis and the function of sleep.

## Introduction

In all mammals, sleep appears to be indispensable and coincides with a conserved circadian temperature rhythm. When our core and brain temperatures are in rapid decline we are most likely to choose to sleep, and if we dissociate from this cycle of body cooling we experience insomnia (Hayward, 1968; Campbell and Broughton, 1994; Lack et al., 2008). Here, we review the evidence that thermoregulatory mechanisms are fundamental to sleep and consider the neuronal circuits that connect these two physiologies. These circuits use the presence of warm microclimates to gate sleep and may enhance circadian body cooling as our first non-rapid eye movement (NREM) bout approaches. The same neurons directly link NREM initiation to body cooling and may explain why transitions from wakefulness to NREM sleep, across the sleep cycle, are immediately followed by a decrease in brain temperature, whilst transitions back to REM or WAKE are accompanied by rewarming (Alföldi et al., 1990; Landolt et al., 1995). The partitioning of brain cooling during NREM sleep and the coordination of the circadian core temperature rhythm are important for effective sleep.

This may have particular consequences for energy homeostasis and could open a window on sleep function.

## Preparation for Sleep is a Thermoregulatory Behaviour

**FIGURE 1:**
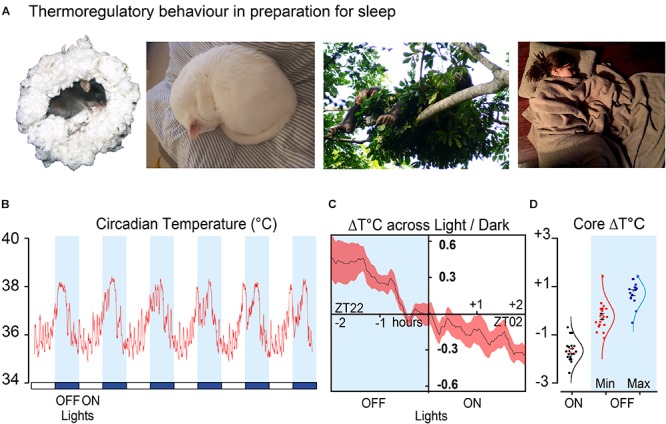
Sleep preparation is a thermoregulatory behaviour **(A)** shows typical nesting behaviour in four species. Mouse nesting (*Mus musculus*, C57Bl6/J), house cat (*Felis catus*) curling up, nest building in the chimpanzee (*Pan troglodytes verus*) and bedding (*Homo sapiens*). **(B)** Example of circadian temperature cycle over 6 days in a male C57Bl6/J mouse. **(C)** Average of transitions from the same mouse over 16 consecutive days 2 h before and after the light change. **(D)** Minimum temperature (*n* = 21) during light phase compared to minimum (*n* = 21) and maximum temperature (*n* = 16) in the dark phase, plotted as change from zero for a group of male C57Bl6/J mice. Data shown in **(B–D)** is from (Harding et al., unpublished). All images used with permission or copyright clearance. The nesting chimpanzee photo credit: Kathelijne Koops. The nesting cat photo credit: Isobel Harding, the sleeping human is available under CC0-1.0 universal and the nesting mouse is adapted from Deacon (2006).

Mammals have a range of thermoregulatory behaviours that allow adaptation to environmental temperature fluctuations throughout the day, but these are most visible in the preparations for sleep (Peever, 2018). These behaviours include warmth and shelter seeking, nest building, curling up and huddling (see Figure 1A). Mice that are inactive or sleeping are much more likely to do so in contact with nesting material (Gaskill et al., 2011). As small rodents they demonstrate surprisingly sophisticated thermal adaptations. As environmental temperature decreases, nest quality rises to compensate and results in measurable improvements in insulation (Gaskill et al., 2013a). When they can, mice engage in huddling with group members (Gaskill et al., 2011, 2012; Gordon et al., 2014). They also have a clear thermal preference during the sleep phase (lights on), choosing warmer environments approaching thermoneutrality (27–30°C) and minimising energy expenditure (Gordon et al., 1998; Gaskill et al., 2012). These behaviours align the circadian temperature decline with the light and dark cycle and sleep onset. An example of the circadian core temperature cycle over several days can be seen in Figure 1B. The decline in core temperature intersects the light–dark cycle and changes over a range of about 2°C at the transition from the active phase of the mice (lights off) to the sleep phase (lights on) (Figure 1C,D).

Ambient temperature is a critical determinant of energy expenditure, and failure to carry out thermal defence behaviours has consequences for energy homeostasis (Yu et al., 2018). Fur removal in Siberian hamsters, for example, increases food consumption by almost a quarter; whereas, in cold conditions, group huddling or the provision of nesting material can reduce food consumption by 15–20%, respectively (Kauffman et al., 2003; Batavia et al., 2010). Similarly, provision of nesting material at temperatures below thermoneutrality increases breeding efficiency giving larger litters, higher pup weight and reduced pup mortality (Gaskill et al., 2013b).

**FIGURE 2:**
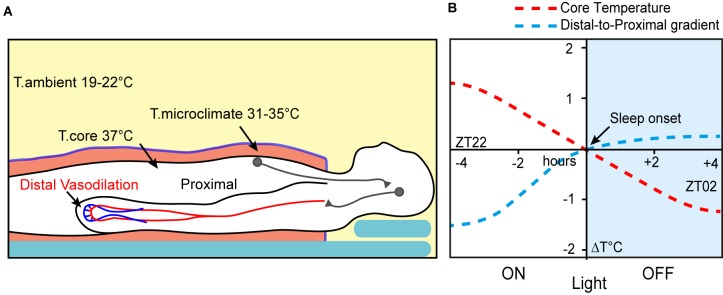
Thermoregulation is important for human sleep. **(A)** Humans use bedding to form warm microclimates during sleep. These activate central hypothalamic mechanisms to induce sleep and peripheral vasodilation. **(B)** Distal-to-proximal gradient and core temperature decline predict sleep onset (adapted from Krauchi et al., 2000).

Thermoregulatory behaviour prior to sleeping is a core part of maintaining energy balance, at least in smaller mammals, where the consequence of thermal inefficiency is an increased need for food. In larger mammals, however, the drive towards thermal preparation for sleep is no weaker. Chimpanzees and other primates select their arboreal sleeping sites (Figure 1A), at least partly, on thermal characteristics, and during colder weather even adjust their nest sites to be more insulating (Koops et al., 2012; Samson and Hunt, 2012; Stewart et al., 2018). In addition, humans actively regulate temperature during sleep by unconsciously increasing their exposed surface area as ambient temperatures rise. In optimal room temperatures, approximately 19–21°C, we attempt to establish skin microclimates between 31 and 35°C and deviation from this range has a negative influence on sleep (Figure 2A) (Muzet et al., 1984; Okamoto-Mizuno et al., 2003; Raymann et al., 2005). A key factor in using microclimates is that, at least in humans, it cannot be replaced by ambient warming at the same temperature, perhaps because it disrupts the self-adjustment required over the course of the night (Muzet et al., 1984; Raymann et al., 2008).

In summary, thermoregulatory behaviours prior to sleep are conserved across mammalian species suggesting they are not just a matter of comfort and may have a more functional role in sleep initiation and maintenance.

## The “Warm Bath Effect”

In humans, immersion in hot water prior to, but not immediately before, the sleep period decreases sleep latency and increases sleep depth. This is the known as the ‘Warm Bath Effect’ (Horne and Reid, 1985; Parmeggiani, 1987; Bunnell et al., 1988; Shapiro et al., 1989; Jordan et al., 1990; Dorsey et al., 1999). In fact, warming for up to 4 h, between 1 and 8 h before to going to bed, increases slow wave sleep (SWS), increases NREM consolidation and decreases REM sleep. This effect embodies a key connection between temperature and sleep. Warming, at the right time, is causatively associated with sleep initiation. However, sleep initiation occurs within the decline of circadian temperature and NREM is associated with further reductions in the temperature of both core and the brain (Alföldi et al., 1990; Landolt et al., 1995; Kräuchi and Wirz-Justice, 2001). Many sleep studies have attempted to reconcile this counter-intuitive relationship to explain two conditions: How warming might initiate sleep and be compatible with body cooling, and how we might encounter this warming under ‘everyday’ conditions.

Optimal ambient temperatures, in combination with bedding, appear to be crucial for efficient sleep onset in humans (Haskell et al., 1981; Okamoto-Mizuno et al., 2003; Raymann et al., 2008). Responses to external temperature also appear to be important as the extent of vasodilation, particularly in the hands and feet (distal-skin), is a good predictor of sleep initiation (Krauchi et al., 1999). This vasodilation is usually considered part of the circadian temperature decline and is observed up to 2 h prior to the start of the first sleep episode, during the wake phase (Krauchi et al., 2000). As core temperature falls it coincides with a decrease in self-assessed alertness (Czeisler et al., 1980; van den Heuvel et al., 1998). In experiments where participants ‘self-selected’ their bed time, subjects were most likely to select a moment when the body temperature was at its maximum rate of decline (Campbell and Broughton, 1994). As sleep approaches, core temperature and heart rate drop, and their steepest decline intersects ‘lights-off’ and sleep onset (Figure 2B). At this point the proximal-to-distal temperature gradient is as much as 1.5°C, but as core temperature falls the gradient reduces to about 0.5°C; a new cooler set-point is reached just after sleep onset. The lowest core temperature is observed about 2 h after ‘lights-off’ and sleep onset in *Homo sapiens* (Krauchi et al., 2000). Under natural conditions, increased circulating melatonin also coincides with declining core temperature prior to sleep onset (Krauchi et al., 1997; Krauchi et al., 2006; Logan and McClung, 2019).

Studying the temperature dependence of sleep in people has always been confounded by our ability to manipulate our environment and escape daily fluctuations in light and temperature. To get around this, Yetish et al. (2015) looked at sleep in three geographically distinct pre-industrial societies. They found that sleep onset coincided most strongly with a reduction in environmental temperature. Sleep was most often initiated after dark and the entire sleep period took place as ambient temperature was declining. Awakening also occurred before dawn, as ambient temperature reached its lowest point, and coincided with vasoconstriction, as measured by finger temperature (Yetish et al., 2015). A change in temperature in the fingers is a good measure of change in blood flow, and so it seems likely that these subjects started sleep in a state of vasodilation that was progressively replaced by vasoconstriction until waking (Rubinstein and Sessler, 1990; van Marken Lichtenbelt et al., 2006). A similar result was also observed by Han et al. (2018), under sleep-laboratory conditions, with high numbers of skin-temperature sensors distributed across the body. These indicated progressive vasodilation from sleep onset to waking. However, this was mostly represented in the torso, and the hands and feet were not recorded (Han et al., 2018).

The circadian cycle and the onset of the first NREM episode are strongly linked. If sleep onset is postponed by sleep deprivation, then the circadian temperature rhythm is disrupted. Similarly, a delay in core temperature decline of more than 2 h, is observed in patients with delayed sleep phase disorders (DSPDs) (Ozaki et al., 1996; van den Heuvel et al., 1998; Watanabe et al., 2003). Disruption of the peripheral vasodilatory response is sufficient to disrupt sleep. For instance, those with difficulty in peripheral vasodilation (vasospastic disorders) have longer sleep latencies than healthy controls (Pache et al., 2001). Narcoleptic patients also have a strongly altered proximal-to-distal skin temperature gradient during daytime waking (Fronczek et al., 2006). But manipulating the proximal-to-distal relationship can change sleep propensity. Warming of the core (proximal-skin) by less than 1°C, easily within the range encountered within the circadian day, is sufficient to shorten sleep latency (Raymann et al., 2005). Temperature manipulation can also selectively and predictably alter vigilance states in patients with narcolepsy (Fronczek et al., 2008a,b). Additional work in the clinic has shown that neonates are three times more likely to fall asleep within 30 min, if their distal-to-proximal skin gradient is greater than 2.5°C (Abe and Kodama, 2015). Distal vasodilation and higher foot temperature in preterm neonates is also correlated with shorter wake bouts (Barcat et al., 2017).

Understanding how warmth might be encountered on a daily basis to precipitate these changes that initiate sleep and vasodilation is crucial. But it seems that the ‘warm bath effect’ is more subtle than previously thought. Raymann et al. (2008) have extended the warming paradigm with the aid of a custom-made ‘thermosuit’ for the manipulation of skin temperature. Small changes in skin temperature of only 0.4°C (in the 31–35 range), can shorten sleep latencies without altering core temperature. They can even encourage deeper sleep in more challenging patient groups, such as elderly insomniacs (Raymann et al., 2008). This latter group was particularly susceptible to this thermal management, supporting the hypothesis that sleep difficulties in the elderly relate to deficits in normal thermoregulation (Raymann and Van Someren, 2008).

In summary, humans and other mammals show thermoregulatory behaviour in preparation for sleep, including curling up, using bedding and nest building. This may generate a microclimate of warmth around the skin that enables entry into sleep while facilitating vasodilation in the ‘distal’ hands and feet. This vasodilation may prepare the ‘proximal’ core for the cooler and inactive phase of the circadian cycle. This warming persists through the night to maintain a sleep-permissive state that also permits selective vasodilation in NREM and constriction in REM and wake. It does so whilst maximising thermal efficiency of the core. The reasons that body cooling and sleep onset coincide are not clear. Body and brain cooling *per se* has not been shown to initiate NREM but is instead a consequence of vasodilation. We might expect that an upstream mechanism in the brain coordinates both NREM onset and vasodilation and in the next section we discuss how this might function (Van Someren, 2000).

## Neuronal Control of Thermogenesis and its Influence on Sleep

**FIGURE 3:**
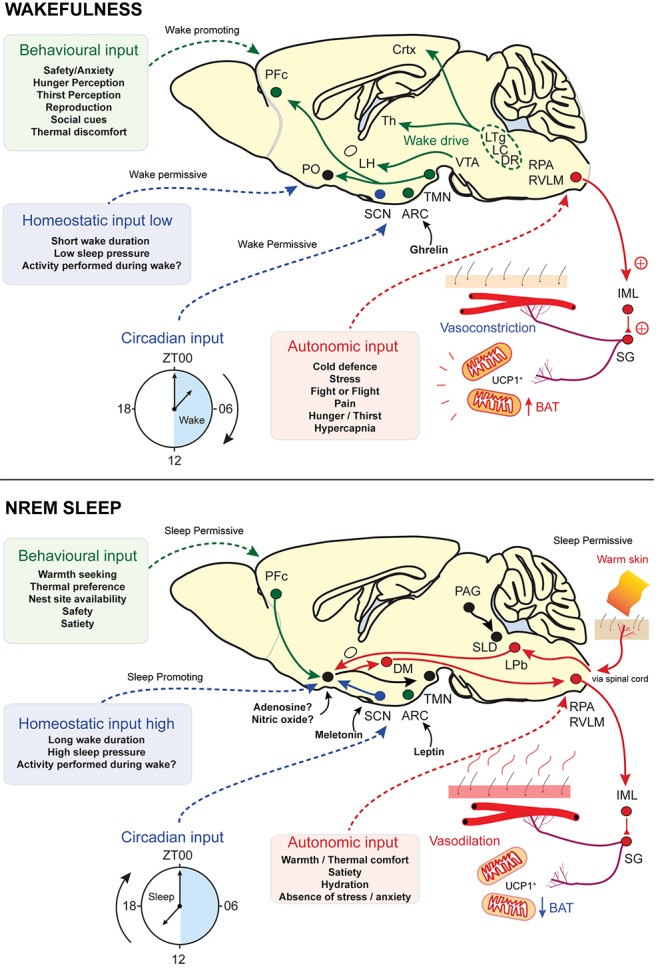
Sensory and homeostatic inputs that gate sleep. Sleep onset is determined by four competing inputs: the homeostatic drive to sleep and three permissive conditions that relate to sleep timing, the behavioural input, the circadian input and the autonomic input. Endocrine inputs are also a key part of each category. Ghrelin and leptin are important for sensing of hunger/satiety, respectively, while melatonin is a key component of the circadian rhythm. Adenosine and NO may form part of the homeostatic input. **(Top - Factors promoting wakefulness)** Circadian cues are permissive for wake and homeostatic pressure to sleep is low. Behavioural factors also promote wakefulness and autonomic inputs are not permissive for sleep. Wake promoting nuclei drive cortical and thalamic excitability, whilst inhibiting sleep-prompting areas such as PO and vPAG. Behavioural needs of food and reproduction overcome those of sleep and thermal comfort. Behavioural inputs are also wake-promoting and may integrate this information in the VTA. Hormonal inputs, such as ghrelin, are detected in the ARC and are sleep-permissive. Autonomic signals, such as ambient temperature, are relayed via the spinal cord and pass through the LPb to the PO for integration. Circuits detecting environmental warmth are not active, vasoconstriction dominates and BAT is active. AgRP neurons signal hunger and inhibit sleep. **(Bottom – Factors promoting NREM sleep)** Circadian cues are now permissive for sleep and homeostatic pressure to sleep is high. Behavioural factors also promote sleep and autonomic inputs are permissive for sleep. On seeking shelter and warmth and having eaten, sleep is permitted. Autonomic signals, such as ambient temperature, are relayed via the spine and pass through the LPb to the PO for integration. NOS1-glutamate neurons are activated by skin warmth and initiate both NREM and body cooling. Activation of vasodilatory and BAT downregulation circuits is via NOS1 projections to LPO GABAergic neurons or via direct projections to DMH and rRPA/RVLM. Behavioural inputs are now sleep promoting and may integrate this information in the VTA. Hormonal inputs, such as leptin, are detected in the ARC and are sleep permissive. POMC neurons detect satiety and are permissive for sleep. NO, nitric oxide; NOS1, nitric oxide synthase-1; PO, preoptic area; LPO, lateral preoptic area; vPAG, ventral periaqueductal grey; TMN, tuberomammillary nucleus; VTA, ventral tegmental area; ARC, arcuate nucleus; LPb, lateral parabrachial; LC, locus coeruleus; DR, dorsal raphe; BAT, brown adipose tissue; AgRP, agouti-related peptide; DMH, dorsal medial hypothalamus; rRPA, rostral raphe pallidus; RVLM, rostral ventrolateral medulla; POMC, pro-opiomelanocortin (Leshan et al., 2012; Eban-Rothschild et al., 2016; Weber and Dan, 2016; Yu et al., 2016; Goldstein et al., 2018; Harding et al., 2018; Yu et al., 2019).

Sleep is a fundamental physiological process that is widely believed to be essential for life but its vital function has yet to be identified. The neuronal circuits that control sleep need to integrate information from at least two distinct inputs. According to current thinking, these are known as Process C and Process S, the circadian and the homeostatic input, respectively, and are part of the two-process model (Borbély, 1982). Transitions from wake to NREM and REM sleep are carried out by neurons that respond to cues from the homeostatic drive, that tracks the time spent awake, as well as more salient cues from the circadian zeitgeber, via the suprachiasmatic nucleus (SCN). The homeostatic process tracks the duration of the waking period and dissipates this load during sleep. However, as we have seen, sleep onset is also gated by other inputs: ambient temperature, as well as levels of satiety, mating opportunities and the need to escape predators all determine the appropriate moment for NREM onset (Borbély, 1982; Borbély et al., 2016; Eban-Rothschild et al., 2017; Logan and McClung, 2019). Neurons that influence sleep are widely distributed throughout the brain. This may allow the integration of behavioural and autonomic inputs onto the classical homeostatic and circadian sleep drive. For example, inhibition of ventral tegmental area (VTA) dopamine neurons promotes both nesting behaviour and sleep initiation (Eban-Rothschild et al., 2016). While the homeostatic drive promotes sleep following prolonged wakefulness, the circadian, the behavioural and the autonomic inputs are permissive conditions for sleep onset (see Figure 3). These four inputs work together to gate sleep.

Although sleep onset and the regulation of sleep transitions may involve multiple nuclei in the brain, one area has been historically associated with NREM onset. The preoptic hypothalamus (PO) is a key site for NREM initiation but is also considered an integrator for thermoregulatory information, including cold and warm-defence (Szymusiak et al., 2007). It consists primarily of the median (MnPO), the medial (MPO) and lateral (LPO) areas that are associated with a large array of functions from sleep to parental behaviour.

Preoptic circuits have been proposed as the mechanistic connection between whole body warming and sleep induction (Morairty et al., 1993). The simplest version of this idea is that warming induces activity in sleep-promoting neurons. Indeed, warm stimuli are well known to increase activity in the PO (e.g., as seen by c-FOS expression) (Scammell et al., 1993; Gong et al., 2000). Consistent with this idea, lesions in the PO of the cat disrupt both warm-defence behaviour and reduce total sleep (Szymusiak et al., 1991). Only significant warming of these cats was able to rescue normal sleep amounts, possibly through compensation or mechanisms outside the PO (Szymusiak and McGinty, 1986). In rats, PO lesions alter thermal preference behaviour which subsequently converges on warmer temperatures (∼30°C) that promote sleep recovery (Ray et al., 2005). In crucial experiments, using a ‘thermode’ implanted into the PO, warming, but not cooling, increases delta power in the EEG (Roberts and Robinson, 1969; Glotzbach and Heller, 1976; McGinty et al., 1994). To characterise preoptic neurons in this role Alam et al. (1995) repeated this protocol using an implanted microdrive and recorded the properties of preoptic neurons. Remarkably, 21% were thermosensitive and these could be further divided into two groups – cold-sensitive neurons (CSNs) and warm-sensitive neurons (WSNs). About 60% of WSN also increased their activity during NREM (Alam et al., 1995). During warming in the rat brain, they could inhibit important arousal nuclei including dorsal raphe and posterior hypothalamic neurons (Krilowicz et al., 1994; Guzmán-Marín et al., 2000; Steininger et al., 2001). In a detailed analysis of MnPO neurons by Suntsova et al. (2002), more than 75% demonstrated properties that may facilitate NREM sleep induction. This included a gradual increase in firing into, and peaking during, NREM sleep and, unexpectedly, even higher firing rates during REM sleep (Suntsova et al., 2002). Mapping of neuronal projections using retrograde and anterograde tracers has confirmed that the MnPO sends dense innervations to wake-promoting regions and is well placed to influence wake-to-sleep transitions by modulation of the lateral preoptic, lateral hypothalamus and dorsal raphe (Uschakov et al., 2007). Lastly, some MnPO neurons express c-FOS in response to sleep deprivation and may also send projections to the LPO (Chou et al., 2002; Zhang et al., 2015).

WSNs can directly sense brain temperature and are proposed to be modulated by pyrogens such as prostaglandin E2 (Scammell et al., 1996; Lazarus et al., 2007). A population of glutamatergic neurons in the midline PO express the transient receptor potential member 2 (TRPM2) channel, enabling direct warm-sensing of local brain temperature. These could function to carry out heat defence but can also modulate the response to fever (Song et al., 2016).

**FIGURE 4:**
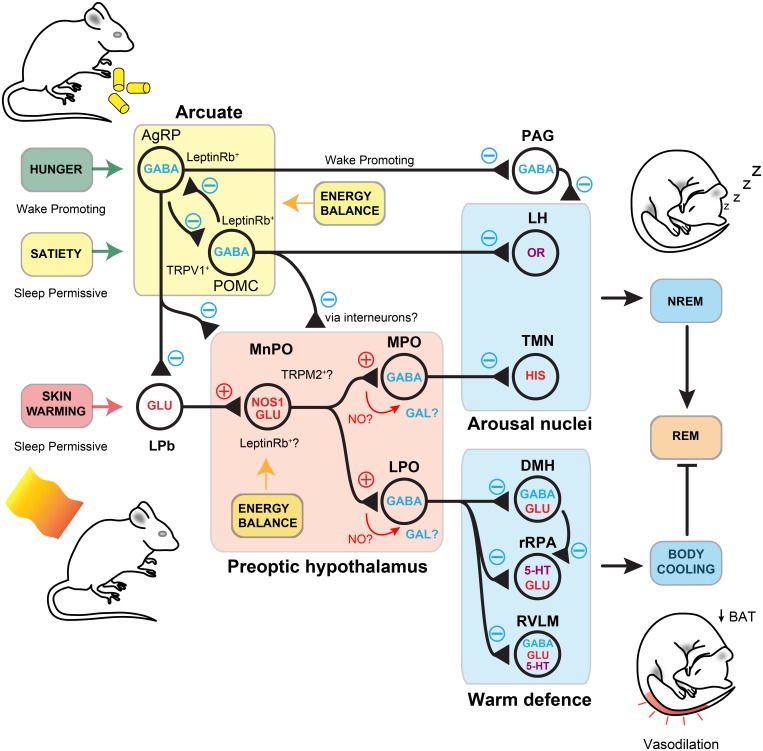
Signal integration in the preoptic hypothalamus. Warmth on the skin stimulates sensory inputs, through the LPb, to preoptic nitrergic-glutamatergic neurons that initiate simultaneous NREM and body cooling. This maybe through activation of separate GABAergic neurons for sleep and hypothermia in the MPO and LPO, but they may also activate galinergic-GABAergic neurons to initiate sleep and body cooling. The synaptic role of NO is unknown in these circuits but potential sites are labelled. NREM is initiated by inhibition of arousal nuclei, including the TMN and the LH. Others are likely to be involved. Body cooling is facilitated by activation of DMH and inhibition of rRPA neurons to induce vasodilation and downregulation of BAT thermogenesis. Inputs to the lateral parabrachial and the preoptic area are modulated by AgRP neuron-mediate inhibition from the arcuate. These detect hunger and put a break on NREM. Satiety induces activation of POMC neurons, that also express TRPV1, are permissive for NREM and induce local inhibition of AgRP neurons. Nitrergic-glutamate neurons may respond to leptin through the leptin Rb, as do AgRP and POMC neurons. They, or a separate local population, may also respond to changes in brain temperature through the TRPM2 ion channel. NO, nitric oxide; NOS1, nitric oxide synthase-1; PO, preoptic area; LPO, lateral preoptic area; vPAG, ventral periaqueductal grey; TMN, tuberomammillary nucleus; ARC, arcuate nucleus; LPb, lateral parabrachial; BAT, brown adipose tissue; AgRP, agouti-related peptide; POMC, pro-opiomelanocortin; DMH, dorsal medial hypothalamus; rRPA, rostral raphe pallidus; RVLM, rostral ventrolateral medulla; TRPM2, transient receptor potential cation channel; TRPV1, transient receptor potential cation channel vallinoid-1; GAL, Galanin (Leshan et al., 2012; Weber and Dan, 2016; Yu et al., 2016; Goldstein et al., 2018; Harding et al., 2018; Jeong et al., 2018; Tan and Knight, 2018; Yu et al., 2019).

With the exception of fever, it is unclear if skin warming could induce an increase in brain temperature that could be sensed by WSNs (Tan et al., 2016; Siemens and Kamm, 2018). Instead, a synaptic pathway is more likely. Neurons that receive afferent temperature information, but are not direct temperature ‘sensors’, have been distinguished by the term ‘warm-activated’ neurons (Tan and Knight, 2018). The MnPO and MPO hypothalamus receives sensory afferents conveying thermal information from the skin (Hammel, 1968; Boulant and Gonzalez, 1977; Morrison and Nakamura, 2011) (Figure 4). Sensory neurons relay ambient temperature information via the spinal cord to glutamatergic relay neurons, and on to sub-regions of the lateral parabrachial nucleus (LPb). The LPb may also receive information from other parts of the body such as the viscera, and then pass these signals onto the MnPO and MPO regions (Nakamura and Morrison, 2008, 2010). At the first point of integration, the glutamatergic neurons conveying excessive warmth synapse at glutamatergic neurons in the MnPO, whose output initiates cooling by promoting vasodilation and switching off brown fat thermogenesis (Morrison and Nakamura, 2011) (Figure 4). What are these neurons in the PO that respond to warming? Recent work using GCaMP6 photometry has shown that these neurons can response to external warm challenges between 30 and 40°C and RNA sequencing has identified them as expressing pituitary adenylate cyclase-activating polypeptide (PACAP) and brain-derived neurotrophic factor (BDNF) (Tan et al., 2016). These neurons are predominantly GABAeric and can induce hypothermia when activated. They function, at least in part, by inhibiting dorsal medial hypothalamus (DMH) glutamatergic neurons that stimulate BAT thermogenesis (Tan et al., 2016). A further population of GABAergic neurons, that act through the DMH, have also been discovered in the nearby ventral LPO (Zhao et al., 2017).

The PO is highly diverse region with many overlapping populations, but only some of these neurons have been functionally characterised (Moffitt et al., 2018). For example, GABAergic-galanin neurons are associated with both sleep and parental behaviour, but populations of galanin-glutamate neurons also exist (Sherin et al., 1998; Wu et al., 2014; Moffitt et al., 2018). PACAP/BDNF, TRPM2-glutamate and nitrergic-glutamate neurons are associated with warm-defence and fever but many other subpopulations exist (Song et al., 2016; Tan et al., 2016; Harding et al., 2018). Whilst the latter is also associated with sleep induction, populations of GABAergic-nitrergic neurons have been found but are uncharacterised (Harding et al., 2018; Moffitt et al., 2018). Given extensive diversity in PO neuronal subtypes (Moffitt et al., 2018), methods such as c-FOS-dependent activity tagging, that allow functional dissection of specific circuits from the surrounding milieu, are particularly important (Zhang et al., 2015). The PO area, including both MPO and LPO responds to recovery sleep, the sleep following sleep deprivation, by expressing c-FOS. The same regions are excited by the, α_2A_-adrenergic agonist and sedative, dexmedetomidine (DEX) (Zhang et al., 2015). To understand whether these physiologies shared the same circuitry Zhang et al. (2015) used c-FOS-dependent activity-tagging to separate the neurons activated by recovery sleep or DEX from other PO neurons that respond to a variety of environmental and homeostatic stimuli. These neurons expressed an excitatory hM_3_d_q_ DREADD receptor such that, when these mice were given clozapine *N*-oxide, only this unique ensemble was activated. This resulted in consolidated NREM sleep, consistent with recovery sleep or sedation. However, PO ensembles, tagged by either recovery sleep or DEX, also induced hypothermia (Zhang et al., 2015). In fact, essentially all sedatives and general anaesthetics used clinically induce core-to-peripheral heat redistribution from vasodilation and, without warming, hypothermia (Díaz and Becker, 2010; Sessler, 2016). This suggests that core PO circuitry could link natural sleep induction, the induction of body cooling and the mechanisms of sedative class drugs.

We hypothesised that thermoregulatory circuits themselves might have an important role in facilitating sleep. This would also explain the propensity of either external or direct PO warming to induce NREM sleep. We again used activity-tagging, but this time labelled only those preoptic ensembles that received warm sensory information. Reactivation of these ‘warm-tagged’ neurons produced simultaneous NREM and body cooling (Harding et al., 2018). These neurons expressed a mixture of cell type markers including the vesicular glutamate transporter 2 (VGLUT2), glutamate decarboxylase (GAD67), and nitric oxide synthase 1 (NOS1). When activity-tagging was repeated in NOS1-CRE mice, these also experienced simultaneous NREM and body cooling. However, when it was repeated in vGAT-CRE mice only NREM and a little body cooling was observed. As these NOS1 neurons express VGLUT2, our data suggest a distinct nitrergic-glutamatergic circuit for linking thermal sensory information to NREM-onset that may reside upstream of a GABAergic sleep ‘switch’ (Harding et al., 2018). In this circuitry, external warmth is a permissive state for NREM initiation. Without this sensory input NREM onset is inhibited. We think this is consistent with data showing that external warming promotes sleep in humans and animals, whilst also providing a possible mechanism for why mammals seek nesting sites: to produce microclimates of skin warmth that permit sleep. We don’t yet know if NOS1 neurons utilise nitric oxide (NO) in synaptic transmission. However, NO is implicated in modulating arousal in other areas of the brain (Gerashchenko et al., 2008; Kalinchuk et al., 2010; Cespuglio et al., 2012; Morairty et al., 2013; Yu et al., 2019).

What is downstream of the MnPO/MPO nitrergic-glutamate neurons? The local preoptic area contains multiple populations of galanin neurons both excitatory and inhibitory (Moffitt et al., 2018). Recently, experiments have demonstrated that activating galanin neurons in the ventrolateral preoptic area (VLPO) can induce both NREM and hypothermia (Kroeger et al., 2018). Similarly, activation of galanin neurons in LPO can also induce NREM and hypothermia (Ma et al., 2019). The latter has parallels to the activation of GABAergic neurons activity-tagged during recovery sleep (Zhang et al., 2015). As the MnPO is known to send projections to both LPO and VLPO we have hypothesised that GABAergic-galanin neurons may be targets for nitrergic-glutamate neurons (Uschakov et al., 2007). In VLPO, activation of galanin neurons using DREADD receptors facilitated more NREM sleep when the mice were closer to thermoneutrality (29°C) and when hypothermia was blunted by warming at 36°C. Thermoneutrality appears to allow optimum recovery of REM sleep, compared to either the ambient (22°C) or warmed (36°C) conditions (Kroeger et al., 2018). This is consistent with the idea of a narrow temperature range for optimised REM sleep (Czeisler et al., 1980; Szymusiak and Satinoff, 1981). Galanin neurons in LPO appear to be necessary for the activation of homeostatic mechanisms that trigger recovery sleep. Deletion of these neurons using caspase expression ablates rebound delta power following sleep deprivation (Ma et al., 2019). More data is needed to confirm if LPO-galanin are genuine targets of nitrergic-glutamate neurons. The latter may also have other long-range projections.

## Thermogenesis Links Sleep to Energy Homeostasis

Thermoregulation, in particular thermal inefficiency, impacts energy homeostasis and changes feeding requirements. This is an additional homeostatic drive that adds its own ‘pressure’ to modulate sleep networks (Figure 3). Following a meal, adipocytes secrete the hormone leptin. This hormone is indicative of excess energy intake and discourages feeding. Leptin works through well-established pathways in the arcuate hypothalamic nucleus, where it inhibits NPY expressing AgRP neurons (Williams et al., 2009). However, there are also leptin receptors elsewhere in the CNS, including in the PO hypothalamus. PO glutamatergic neurons expressing the leptin receptor (leptinRb) are excited (they express c-FOS) when ambient temperature rises (Yu et al., 2016). This results in a reduced energy expenditure, through inhibited thermogenesis, and a decrease in food consumption (Zhang et al., 2011; Yu et al., 2016). Neurons that coexpress NOS1 and leptinRb have been identified in other parts of the hypothalamus and these can also inhibit thermogenesis (Leshan et al., 2012). Hence, it seems likely that there is some overlap between preoptic glutamatergic-leptinRb neurons and the NOS1 populations identified in Harding et al. (2018). Similarly, many BDNF/PACAP in the PO express VGLUT2 and a subpopulation of these neurons that express c-FOS in response to a warm stimulus has recently been shown to coexpress the leptinRb suggesting yet further overlap between these populations (Moffitt et al., 2018). As well as energy regulation, leptin signalling appears to have a more direct role in sleep. Systemic administration of recombinant leptin in food-deprived mice increases both NREM and REM sleep durations, while mice deficient in leptin (ob/ob) have fragmented sleep as well as lower average core temperature (Sinton et al., 1999; Laposky et al., 2006). A key remaining question is whether NOS1 neurons that initiate NREM sleep and body cooling provide a wider link between sleep and energy homeostasis (Harding et al., 2018). These circuits are summarised in Figure 4.

Recent data have provided a new insight into how energy balance might influence sleep. Goldstein et al. (2018) have directly assessed the impact of AgRP/POMC neuronal activity in the arcuate nucleus on the drive to sleep. AgRP neurons can detect energy intake and are considered ‘hunger sensors’, inhibited by both circulating leptin and insulin. POMC oppose the action of AgRP neurons and are activated by leptin (Cowley et al., 2001). AgRP activity promotes food seeking behaviour, even at the expense of sleep. But, if mice are food deprived, inhibition of these neurons rescues sleeping behaviour at the expense of eating (Goldstein et al., 2018). As thermal inefficiency results in increased feeding, we would expect circuitry that links thermal sensation to appetite control. Consistent with this idea, Jeong et al. (2018) have shown that POMC neurons express the transient receptor potential vanilloid-1 (TRPV1) channel. Optogenetic activation of these neurons produces feeding inhibition (Jeong et al., 2018). Although this study did not assess sleep, activation of POMC neurons by Goldstein et al. (2018) could rescue sleep in animals deprived of food (Goldstein et al., 2018). This may be because POMC neurons densely innervate sleep-promoting areas including the PO and may inhibit local GABAergic interneurons (Elias et al., 1999; Wang et al., 2015; Weber and Dan, 2016). POMC neurons also inhibit AgRP neurons which project to several sleep-promoting regions including the PO, the ventral periaqueductal grey (vPAG) and parabrachial nucleus (Pb) (Betley et al., 2013; Wang et al., 2015; Weber and Dan, 2016; Weber et al., 2018) (see Figure 4). Arcuate NPY neurons that normally stimulate eating also downregulate BAT thermogenesis and so may have similar roles in thermoregulatory connections to sleep (Shi et al., 2013). These autonomic signals are interpreted as strong behavioural drives, for instance to find food.

In summary, it seems likely that there is significant overlap between neuronal populations that regulate sleep onset, thermogenesis and energy homeostasis. Sleep onset may be controlled, in part, by integrating these sensory inputs, including ambient temperature and energy status. It is less clear why gating sleep with these inputs would be beneficial.

## Sleep Deprivation Disrupts Thermoregulation and Energy Balance

Sleep architecture is highly dependent on thermal factors, but the consequence of total sleep loss is a radical alteration of thermoregulation and energy balance. In rats, chronic total-sleep deprivation and selective REM deprivation, using the disk-over-water method for many days, leads to profound physiological effects and eventually death (Everson et al., 1989). In the early stages, an increase in metabolic function was observed in these rats, including core body temperature, and with it an increase in food consumption. However, the temperature rise was quickly reversed and the rats progressively developed hypothermia. They also moved to warmer parts of a temperature gradient as their sleep deprivation deepened (Prete et al., 1991). This may be an energy conservation strategy, reducing thermal load, increasing appetite and simultaneously cooling the body (Rechtschaffen and Bergmann, 1995). Similar strategies are seen in torpid animals when food is scarce (Ruf and Geiser, 2015). In the sleep-deprived animals this strategy ultimately failed, as the rats rapidly lost weight (Bergmann et al., 1989; Everson et al., 1989; Rechtschaffen and Bergmann, 1995). Sleep deprivation appears to either increase the metabolic requirements of the animal, or by other means facilitates excessive heat loss, perhaps through over activation of NREM-initiating circuits that induce vasodilation.

One mechanism by which mammals, and small rodents in particular, generate heat is through brown adipose tissue thermogenesis (BAT). This is also a key mechanism in regulating energy homeostasis. Uncoupling protein 1 (UCP-1) is a key component of thermogenesis in brown adipose tissue (BAT). It decouples the electron transport chain from the ATP-synthase, facilitating heat production through proton gradient dissipation, without ATP production, and compensatory metabolism (Cannon and Nedergaard, 2004). UCP-1 knockout mice have weakened homeostatic rebound following sleep deprivation. They also show a blunted sleep induction effect of warmer temperatures observed in control mice (Szentirmai and Kapas, 2014). Similarly, the β3-adrenergic receptor agonists, which activate BAT thermogenesis, usually induce sleep in control mice but this response is ablated in mice with chemical deafferentation of the BAT (Szentirmai and Kapás, 2017). These data suggest that UCP-1 mediated BAT thermogenesis is helpful in both recovery sleep (sleep following sleep deprivation) and NREM sleep induction. UCP-1 may also have a role facilitating NREM sleep during systemic inflammation (Szentirmai and Kapas, 2018). The heat generated by these mechanisms could activate the sensory receptors in the skin and so trigger NREM sleep (Harding et al., 2018).

## Torpor and Hibernation: Too Cold to Sleep?

**FIGURE 5:**
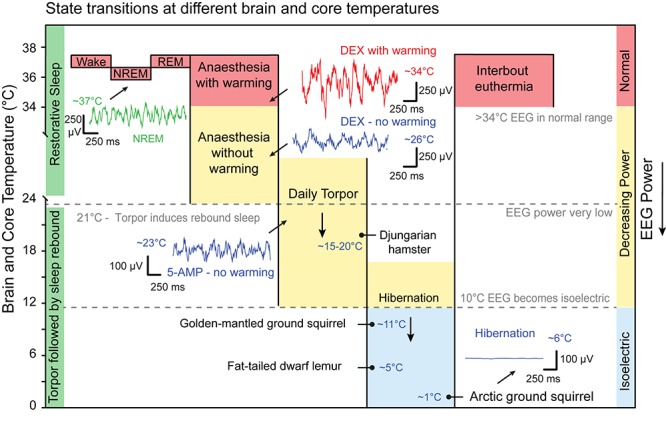
State transitions at different core temperatures. Sleep, anaesthesia and torpor sit on a continuum of decreasing core temperature that directly influences EEG power. On average NREM bouts are cooler than those of wake whilst brain temperature during REM is warmer. A representative example of NREM EEG at approximately 37°C is shown in green. Sedatives and anaesthetics induces delta oscillations in the EEG but also hypothermia. Dexmedetomidine (DEX; 100 μg/kg IP) induces sustained sedation but the power of the delta oscillations is suppressed. The example is shown in blue, 2 h after injection with core temperature at approximately 26°C. If the same dose of DEX is given to animal in a warm chamber then the power of the delta oscillations recovers. The example is shown in red 2 h after injection with a core temperature at 34°C. Some mammals use daily torpor to save energy during times of food scarcity. On average these are between 15 and 20°C but can range between 10 and 30°C. At approximately 21°C states of torpor may generate a sleep debt that results in recovery sleep on rewarming to 37°C. Artificial hypothermia, sometimes known as synthetic torpor, can be induced by 5-AMP (0.5 g/kg IP). This also induces delta oscillations that are suppressed by hypothermia. The example is shown in blue, 1.5 h after injection with core temperature at approximately 23°C. Below approximately 10°C the EEG is isoelectric and no oscillation can be discerned. Hibernators have periods have interbout euthermia with normal EEG power and wake-NREM and wake-REM transitions are detected. Example species are labelled with the temperature that they have been observed in for either daily torpor or hibernation. This reflects ambient environmental conditions important for EEG measurements but is not a strict hierarchy. EEG examples are from (Harding et al., unpublished) except for the hibernation example which is adapted from Frerichs et al. (1994). IP, intraperitoneal; 5-AMP, adenosine monophosphate. Djungarian hamster (*Phodopus sungorus*), Golden-mantled ground squirrel (*Callospermophilus lateralis*), Fat-tailed dwarf lemur (*Cheirogaleus medius*), Arctic ground squirrel (*Urocitellus parryii*). Data adapted from Frerichs et al. (1994); Ruf and Geiser (2015), and Vyazovskiy et al. (2017).

The cooperation of body cooling and NREM sleep suggest that energy homeostasis is an important factor for sleep, but it is natural to ask if there is any link to more extreme states (Figure 5). When the need to save energy is sufficiently high many mammals sacrifice sleep to adopt an alternative thermoregulatory strategy of daily torpor or seasonal hibernation (Ruf and Geiser, 2015). Daily torpor is a state of hypothermia triggered by food scarcity. Mammals that use daily torpor, such as the Djungarian hamsters (*Phodopus sungorus*), typically drop their core temperature to 15–20°C for many hours, but, in many species, daily torpor can range between 10°C or as high as 30°C (Ruf and Geiser, 2015) (Figure 5).

A range of mammals from ground squirrels to brown bears also use annual hibernation strategies for winter survival and reproduction (Carey et al., 2003; Ruf and Geiser, 2015). In hibernators core temperatures typically fall between 0 and 10°C and are maintained for weeks or months. Exposed ambient temperatures can fall below 0°C and core temperatures are maintained only a few degrees higher at 1% of euthermic (normal temperature) metabolic rates (Carey et al., 2003). In extreme cases, such as the arctic ground squirrel (*Urocitellus parryii*), abdominal and peripheral temperatures can be stable at around -2°C whilst head and neck are just above 0°C (Barnes, 1989; Boyer and Barnes, 1999). Only at body temperatures around 0°C do metabolic rates rise to defend the animal from freezing, suggesting an extremely low temperature set point (Buck and Barnes, 2000) (Figure 5).

Animals in either daily torpor or hibernation enter a state of inactivity or quiescence, but the power of the EEG signal observed in these animals scales with temperature: the lower the body temperature, the lower the power of the EEG (Deboer, 1998). At a core temperatures of approximately 22°C or above, the frequency component remains within the delta band of 1–4 Hz and can be classified as sleep, although the EEG power is much reduced (Walker et al., 1981; Daan et al., 1991). However, at lower temperatures this is not the case. If core and brain temperature is sufficiently low, then the EEG power falls below the threshold for attribution of sleep states (Figure 5). At brain temperatures between 10°C and 25°C reduced-power delta oscillations can still be identified in the EEG signal, whereas below about 10°C the signal is isoelectric (Walker et al., 1977). It is not clear to what extent the power of these oscillations is important for natural sleep function. In dwarf lemurs (*Cheirogaleus medius*), for example, hibernating at low ambient temperatures of only 5°C, EEG recordings are isoelectric and evidence of NREM or REM sleep are absent. In this case, only on rewarming was sleep (REM) observed (Krystal et al., 2013). It has been suggested that torpor states may be a form of sleep deprivation. For instance, the recovery of Djungarian hamsters from daily torpor, with core and brain temperatures of around 23°C, results in a period of recovery sleep with an increased power in the delta band (Deboer, 1998). Similarly, this sleep could be deferred by sleep deprivation suggesting the accumulation of a sleep debt during torpor (Deboer and Tobler, 1994; Palchykova et al., 2002). While this sleep debt, as measured by delta power, accumulates during torpor it does so almost three times slower at brain temperature below 27°C, compared with time awake (Deboer and Tobler, 2003). However, comparisons of recovery sleep EEG, following sleep deprivation or torpor, revealed differences in cortical network activity suggesting that torpor is not entirely equivalent to either sleep deprivation or natural sleep (Vyazovskiy et al., 2017). Thus, a critical temperature may exist below which sleep function is impaired.

To understand the relationship between sleep and temperature in hibernators, researchers have compared animals that hibernate at different ambient temperatures. Animals that hibernate at low temperatures, such as the arctic ground squirrels (*Urocitellus parryii*), briefly warm up to levels comparable to waking (36–37°C). These are periods of interbout euthermia (Boyer and Barnes, 1999; Carey et al., 2003). In these periods of warming, squirrels transition from wake to NREM and then REM sleep before returning to hibernation (Daan et al., 1991). Hibernation of the golden-mantled ground squirrel (*Callospermophilus lateralis*) under warmer laboratory conditions (22°C) produced continuous NREM sleep (Walker et al., 1981). During hibernation at colder temperatures, of 11°C ambient, the minimum brain temperature, not the hibernation bout length, was the best predictor of rebound delta power during subsequent interbout euthermia. The same authors observed that, at this temperature (11°C), the euthermic (36–37°C) period following hibernation consisted of almost 70–80% NREM sleep, whereas animals hibernating at 21°C spent only 40% of their euthermic period in NREM (Larkin and Heller, 1996). This indicates that the temperature at which hibernation takes places influences the degree to which sleep debt accumulates (see Figure 5). Of course, there are variations between species. When European ground squirrels enter a euthermic period, following hibernation at 5.5°C, the time spent in NREM sleep is proportional to the hibernation bout length, not temperature *per se* (Strijkstra and Daan, 1997). Collectively, these data suggest that the restorative component of sleep is temperature-dependent.

The same temperature dependence of sleep is seen in hibernating primates. When dwarf lemurs (*Cheirogaleus medius*) choose a hibernaculum at warmer temperatures, their EEG resembles NREM and REM sleep and the episodes of euthermia disappear (Dausmann et al., 2004; Krystal et al., 2013). In other species of lemur (*C. crossleyi and C. sibreei*) that occupy a cooler environmental niche, sleep is consistently absent during the torpor phase, but returns during periods of interbout euthermia (Blanco et al., 2016). Black bears (*Ursus americanus*), which always hibernate at warmer temperatures of 32–34°C, and actively defend this temperature set point, also do not show the inter-bout arousals seen in smaller mammals (Tøien et al., 2011). Like the dwarf lemurs, these higher temperatures appear to allow brown bears to spend large amount of time in NREM sleep (Tøien et al., 2015). The defending of lower temperature set points in larger mammals has remarkable parallels with people under anaesthesia. In people given either the sedative DEX or the anaesthetic propofol, shivering thresholds reduce to between 32 and 34°C, respectively (Matsukawa et al., 1995; Talke et al., 1997; Sessler, 2016). Hence, hibernation may be too cold to facilitate sleep and episodes of interbout euthermia, lasting 12–24 h, may allow sleep processes to be recovered (Carey et al., 2003).

The neuronal circuitry that induces torpor and/or hibernation is not known. However, it is possible that it uses components of the natural sleep–wake circuitry. For example, NOS1-glutamate neurons in PO that induce NREM sleep and sustained hypothermia (Harding et al., 2018), could in colder climates, have a role in torpor or hibernation induction, but with different behavioural and environmental triggers.

In summary, NREM sleep in a state of mild body cooling may be the preferred biological condition, but clearly in extreme environments, winter survival or times of food scarcity the restorative effects of sleep are, at least in part, sacrificed for energy conservation. As sleep can only be maintained at higher temperatures, it is energetically more expensive than torpor or hibernation. At these colder brain and core temperatures, sleep debts accumulate almost three times slower than during waking (Deboer and Tobler, 2003).

## Why Link NREM Sleep and Body Cooling?

To recap, sleep in rodents is associated with temperature-cycling: wake to NREM sleep transitions coincide with a cooler body and brain facilitated by tail vasodilation. Indeed, effective thermoregulation and nesting behaviour produce warm microclimates that have a role in stimulating NREM sleep and body cooling. We have suggested that PO neurons both receive warm thermal information from the skin and simultaneously coordinate NREM sleep initiation and body cooling (Harding et al., 2018). Transitions to wakefulness or REM sleep are accompanied by vasoconstriction and brain warming (Alföldi et al., 1990; Imeri and Opp, 2009). The absolute change in brain temperature at each NREM transition is small, about 0.2–0.4°C, but may reach larger values, comparable with the total diurnal variation in temperature (approximately 2°C), during extended bouts of sleep. In humans, core temperature reliably falls about 2 h prior to sleep onset and the first NREM episode is more likely to occur at the steepest point of temperature decline. Brain temperature appears to do the same (Landolt et al., 1995). This rate of decline may be highest when PO circuitry is maximally activated, facilitating NREM sleep to the greatest extent. Other sensory inputs, such as satiety, are also permissive for sleep and their inputs are integrated to determine the precise moment of NREM onset. These temperature changes may have a direct role in the restorative functions of sleep.

One of the first hypothesis regarding the lower temperatures coinciding with NREM sleep was that it existed specifically to cool the brain (McGinty and Szymusiak, 1990). It was proposed that a lower brain temperature would reduce cerebral metabolism, conserve energy and assist other functions from immune regulation to circadian coordination (McGinty and Szymusiak, 1990). Conservation of energy for sleep in its entirety has also been proposed (Berger and Phillips, 1995). However, we have seen that when mammals of all sizes prioritise conservation of energy, extremes of hypometabolism in torpor and hibernation are selected at the expense of sleep (Ruf and Geiser, 2015). This suggests that energy conservation alone is not the primary function of sleep. Indeed, estimates of energy use over 24 h put the cost of sleep as high as 85–95% of the metabolic cost of waking (Jung et al., 2011; Abreu-Vieira et al., 2015; Hibi et al., 2017).

It is feasible that reduced temperatures have a more direct function in the brain. At temperatures of 20°C or less, during which sleep debt is accumulated, morphological changes have been observed in dendritic spines (Peretti et al., 2015). Hibernators can undergo synaptic remodelling while cold, as do animals in artificial torpor induced by 5′-adenosine monophosphate (Popov and Bocharova, 1992; Magariños et al., 2006; Popov et al., 2007). In the latter condition, the total number of synapses is reduced (GM). The presence of these process may explain why sleep, as a restorative process, is inhibited at lower temperatures. Large changes in gene expression are also observed both in the brain and across the body in hibernators (Williams et al., 2005). Colder temperatures, particularly in the brain, can induce expression of so called ‘cold-shock’ proteins including cold-inducible RNA binding protein (CIRP) and RNA-binding motif protein 3 (RBM3) (Morf et al., 2012; Peretti et al., 2015; Hoekstra et al., 2019). Body and brain cooling during natural sleep are small, both from the reduction in diurnal core temperature and reductions in brain temperature at each NREM transition, but recent data suggest they are sufficient to increase CIRP expression and so influence the expression of other genes, including the circadian genes Period and Clock (Morf et al., 2012; Hoekstra et al., 2019). This is important as cortical temperature changes are heavily influenced by the sleep–wake transitions and entry into NREM sleep can then influence clock gene expression to drive further transcriptional changes. In mice without CIRP, sleep deprivation results in 50% less REM sleep, illustrating the strength of this mechanism (Hoekstra et al., 2019). This also provides one possible mechanism that the brain may keep track of the time spent in NREM sleep.

The extensive neuronal inter-connections that cross-regulate energy use, sleep induction and body temperature (see Figure 3) hint that the function of sleep plays an important role in energy homeostasis. The temperature-dependence of sleep debt accumulation, which is slowed at cooler temperatures, suggests that this debt is inherently a metabolic processes. Lastly, the synchronised changes in brain temperature during sleep may coordinate gene expression important for the functions of sleep, whilst contributing to a mechanism that measures the time spent sleeping.

## Author Contributions

EH wrote the manuscript and designed the figures. All authors have discussed and edited the manuscript.

## Conflict of Interest Statement

The authors declare that the research was conducted in the absence of any commercial or financial relationships that could be construed as a potential conflict of interest.
